# miRNA-210 expression is associated with iron deficiency and biochemical parameters in hemodialysis patients

**DOI:** 10.3389/fmed.2026.1668328

**Published:** 2026-03-02

**Authors:** Merve Kılıç, Hamad Dheir, Mahmud İslam, Zafer Ercan, Muhittin Abdulkadir Serdar

**Affiliations:** 1 Department of Biochemistry and Molecular Biology, Graduate School of Health Sciences, Acibadem Mehmet Ali Aydinlar University, Istanbul, Türkiye; 2 Department of Anesthesia Program, Advanced Vocational School, Dogus University, Istanbul, Türkiye; 3 Department of Nephrology, Sakarya University Faculty of Medicine, Sakarya, Türkiye; 4 Department of Nephrology, Sakarya University Training and Research Hospital, Sakarya, Türkiye; 5 Department of Medical Biochemistry, School of Medicine, Acibadem Mehmet Ali Aydinlar University, Istanbul, Türkiye

**Keywords:** miRNA-210, hypoxia, hemodialysis patient, iron deficiency anemia, functional iron deficiency

## Abstract

**Background:**

This study aimed to evaluate the potential of microRNA (miRNA)-210 as a biomarker for distinguishing iron deficiency anemia (IDA) from functional iron deficiency (FID) in hemodialysis (HD) patients. The diagnostic performance of miRNA-210 was also compared with conventional biochemical markers, including hemoglobin (Hb), ferritin, transferrin saturation (TSAT), and zinc protoporphyrin (ZnPP).

**Methods:**

Fifty HD patients were classified into control, IDA, and FID groups according to Hb, ferritin, and TSAT criteria. Pre-dialysis blood samples were collected, and plasma miRNA-210 levels were measured using reverse transcription quantitative polymerase chain reaction (RT^2^-PCR). Diagnostic performance was assessed through receiver operating characteristic (ROC) analysis alongside traditional biomarkers.

**Results:**

Plasma miRNA-210 levels were significantly higher in the IDA group compared to both the control (*p* = 0.0010) and FID (*p* = 0.0007) groups. A significant negative correlation was observed between miRNA-210 and Hb (*ρ* = −0.363, *p* = 0.0155). ROC analysis showed that miRNA-210 had moderate diagnostic discriminatory ability for differentiating IDA (AUC = 0.711, *p* = 0.0186). Its performance was comparable to ZnPP and exceeded to ferritin and TSAT.

**Conclusion:**

miRNA-210 may serve as a supportive biomarker, reflecting the interaction between hypoxia and iron metabolism in distinguishing IDA from FID among HD patients. These findings indicate that miRNA-210 could provide additional value in understanding anemia pathophysiology and enhance diagnostic evaluation.

**Limitations:**

Key limitations include the small sample size, single-center, cross-sectional design, absence of a healthy control group, and lack of molecular-level functional validation. Larger multicenter studies are needed to confirm these findings and determine clinically relevant cut-off values for miRNA-210.

## 1 Introduction

Iron deficiency anemia (IDA) is one of the most common complications in hemodialysis (HD) patients and can negatively impact their quality of life and impair their clinical outcomes. IDA is generally characterized by low hemoglobin (Hb) levels, decreased transferrin saturation (TSAT), and low ferritin levels, which lead to hypoxia, exacerbating the progression of kidney disease and other comorbid diseases (1, 2).

Anemia is a clinical condition characterized by a Hb level of <13 g/dL in men and <12 g/dL in women, leading to a reduction in the capacity to carry oxygen to tissues. Anemia in chronic kidney failure is associated with decreased quality of life, cardiovascular diseases, left ventricular hypertrophy, increased hospitalization rates, and mortality (3, 4). Functional iron deficiency is defined as a serum ferritin level of ≥30 ng/mL but a TSAT of <20% (5).

As chronic kidney disease (CKD) reaches end-stage kidney disease, repeated dialysis becomes a fundamental requirement for survival. These patients frequently undergo HD to manage metabolic waste and fluid imbalances and often develop iron deficiency and anemia due to decreased erythropoiesis, blood loss during dialysis, and iron utilization disorders (3, 6, 7).

Hypoxia is one of the most important factors in the pathogenesis of IDA. Iron plays a critical role in oxygen transport, and its deficiency reduces the capacity to carry oxygen to tissues, leading to hypoxia. This leads to the activation of regulatory mechanisms such as erythropoiesis and the modulation of microRNA (miRNA) expression. miRNAs are small non-coding RNA molecules that play important roles in regulating various physiological processes in response to hypoxia (8–10).

miRNA-210 is known as a miRNA that plays a role in cellular adaptation to low oxygen conditions. This miRNA is particularly effective in regulating critical genes such as angiogenesis, cell proliferation, and apoptosis (8, 10, 11).

Recent studies have suggested that miRNA-210 expression may be altered in IDA patients in the context of hypoxia caused by low Hb levels (12). However, there is a limited number of studies examining how miRNA-210 expression specifically changes in HD patients due to IDA. Therefore, this study aims to examine the differences in miR-210 expression levels between the groups of HD patients defined by Hb, TSAT, and ferritin thresholds and to contribute to the literature.

## 2 Methods

### 2.1 Study population

This study included a group of 50 patients who were undergoing a chronic HD program at the Nephrology Clinic of Sakarya University Training and Research Hospital and were regularly followed between September 1 and December 1, 2023. The study was approved by Acibadem Mehmet Ali Aydinlar University ATADEK (Acıbadem University and Acıbadem Healthcare Organizations Medical Research Ethics Committee) on June 16, 2023, with decision number 2023-10/340. Inclusion criteria included: presenting to the Nephrology Outpatient Clinic between the specified dates, being over 18 years of age, having been diagnosed with CKD at the relevant outpatient clinic, falling outside the exclusion criteria specified in the study plan, signing the consent form, and being on chronic HD program for at least 90 days and 3 days per week. Exclusion criteria included: pregnancy/postpartum/breastfeeding mothers, unconscious individuals, individuals who could not give consent personally, the presence of any active infection, patients with malabsorption syndrome, diagnosed with malignancy, liver disease, diagnosed with hematological disease, active bleeding, history of bleeding in the last 3 months, patients using anticoagulants such as warfarin, and patients enrolled in a diet or drug study.

According to the results of the study by Özdemir et al. (13), the effect size was determined to be 1.03. Based on this, with an alpha value of 5% and a power value of 90%, an *a priori* power analysis was conducted using the G*Power software. The analysis indicated that a minimum sample size of 42 participants, with 21 individuals in each group, was required for the study. Considering potential issues that might arise during the study, it was initiated with 50 participants. After the completion of the study, a *post-hoc* power analysis was performed using the actual sample size, and the statistical power achieved was determined to be 84%.

### 2.2 Collection and storage of analysis samples

Blood samples were collected from patients at one time at the Nephrology Clinic of Sakarya Training and Research Hospital, following a 12-h hunger, and collected in vacuum tubes containing 15 mg/mL ethylenediaminetetraacetic acid (EDTA) anticoagulant before HD. All blood samples were collected immediately before the initiation of the dialysis session to minimize the effect of potential dialysis-induced clearance on circulation miRNA levels. Measurements of the analyzed samples were performed using BC MINDRAY 6200 and BC MINDRAY 6000 devices in the Biochemistry Laboratory of Sakarya Training and Research Hospital. All determined parameters were determined following daily quality control procedures. Plasma was obtained by centrifugation at 3,000 rpm for 10 min, and samples used outside of daily analyses were stored at −80 °C.

### 2.3 miRNA-210 gene expression analysis

The real-time Reverse Transcription Polymerase Chain Reaction (RT-qPCR) method was used to determine miRNA expression levels. Total RNA was purified using an RNA isolation kit (QIAGEN, miRNeasy serum/plasma kit catalog no: 217184) from the manufacturer. Purity measurements of isolated RNAs were performed using a nanodrop spectrometer, and samples with an A260/280 ratio below 1.8–2.0 were excluded from the study. After RNA purification, a cDNA synthesis kit (QIAGEN, miRCURY LNA RT kit catalog no: 339340) was used. A QIAGEN RotorGene device was used for RT-qPCR analyses, and gene expression analysis was performed using relevant kits (QIAGEN miRCURY LNA SYBR Green PCR kit, catalog number: 339345, and QIAGEN miRCURY LNA PCR Assay kit, catalog number: 339306). Normalization in the study was provided with the UniSp6 miRCURY LNA miRNA PCR Assay.

### 2.4 Plasma miRNA isolation protocol

Plasma samples were stored at −80 °C and thawed at room temperature prior to analysis. A total of 100 μL of thawed plasma was mixed with 500 μL of QIAzol lysis solution by pipetting and incubated for 5 min at room temperature. Subsequently, 100 μL of chloroform was added, and the mixture was vortexed vigorously for 15 s, allowed to stand for 2–3 min, and then centrifuged at 12,000 g for 15 min at 4 °C. Following centrifugation, three phases were formed, and the upper, colorless aqueous phase containing RNA was carefully transferred to a new tube. An additional 1.5 volumes of 100% ethanol was added to the aqueous phase, mixed thoroughly, and applied to an RNeasy MinElute spin column. The column was centrifuged at 11,000 rpm for 15 s, followed by sequential washes with 700 μL Buffer RWT, 500 μL Buffer RPE, and 500 μL of 80% ethanol. After the final wash, the column was placed into a new collection tube and centrifuged at maximum speed for 5 min with the lid open to allow drying. For RNA elution, 14 μL of RNase-free water was added directly to the center of the membrane and centrifuged at 11,000 rpm for 1 min, yielding a total of 12 μL of eluate. RNA concentrations were measured using a Nanodrop spectrophotometer, and only samples with an A260/280 ratio between 1.8 and 2.0 were included in the study.

### 2.5 cDNA synthesis

cDNA synthesis was performed using the QIAGEN miRCURY LNA RT Kit (catalog no: 339340). RNA samples were diluted with RNase-free water to a final concentration of 5 ng/μL. Each 20 μL reaction mixture consisted of 4 μL 5 × miRCURY SYBR Green RT Reaction Buffer, 9 μL RNase-free water, 2 μL 10 × miRCURY RT Enzyme Mix, 1 μL UniSp6 RNA spike-in, and 4 μL RNA. The reaction mixtures were placed into PCR strip tubes and processed in a thermal cycler under the following program: incubation at 42 °C for 60 min, followed by enzyme inactivation at 95 °C for 5 min and rapid cooling to 4 °C. The resulting cDNA was stored and subsequently used for real-time PCR analysis.

### 2.6 Real-time PCR analysis

RT-qPCR analyses were performed using the QIAGEN Rotor-Gene real-time PCR system. Each reaction had a final volume of 20 μL, consisting of 10 μL 2 × miRCURY SYBR Green Master Mix, 2 μL PCR Primer Mix, 2 μL RNase-free water, and 4 μL diluted cDNA. The PCR cycling conditions included an initial denaturation at 95 °C for 2 min, followed by 40 cycles of 95 °C for 10 s and 56 °C for 1 min. Ct values were automatically generated by the system software. Melt-curve analysis was performed to confirm the specificity of the amplified products.

### 2.7 Normalization strategy

Because universally accepted endogenous reference miRNAs are lacking in plasma and serum samples, normalization was carried out using the UniSp6 spike-in control (QIAGEN miRCURY LNA miRNA PCR Assay, catalog no: 339306) to control for technical variability during RNA extraction and reverse transcription steps. Relative expression levels were calculated using the *ΔΔCt* method, where *ΔCt* = Ct_miR-210 – Ct_UniSp6. Fold changes were then calculated using the 2^-*ΔΔCt* method. *ΔΔCt* was obtained by subtracting the mean *ΔCt* value of the control group from the mean *ΔCt* value of the patient group. A fold change greater than 2 was interpreted as upregulation of expression, while a value less than 1 was considered downregulation. Reactions with a Ct value greater than 35 or a replicate standard deviation (SD) above 0.5 were excluded from the analysis. Inter-run calibrators were used to minimize plate-to-plate variability, and melt-curve analysis confirmed the presence of single, specific amplification products.

### 2.8 Data analysis

Quantification of miRNA expression was normalized using the UniSp6 spike-in reference gene. *ΔCt* and *ΔΔCt* values were used to calculate fold change, and results were subjected to statistical analysis. All assay performance characteristics are summarized in Table 1.

**Table 1:** Real-time PCR assay details.

Target miRNA	Assay ID/catalog no.	Amplicon length (bp)	qPCR kit (catalog no.)	Instrument	Efficiency (%)	*R*^2^ (standard curve)
miR-210	Qiagen miRCURY LNA PCR Assay, 339,306	72	Qiagen miRCURY LNA SYBR Green PCR Kit, 339,345	QIAGEN Rotor-Gene	96.5	0.998
UniSp6 *(spike-in control)*	Qiagen miRCURY LNA PCR Assay, 339,306	68	Qiagen miRCURY LNA SYBR Green PCR Kit, 339,345	QIAGEN Rotor-Gene	94.2	0.997

## 3 Results

### 3.1 Demographic findings and distributions of HD patients

This study included 50 chronic HD patients receiving treatment at the Nephrology Clinic of Sakarya Training and Research Hospital. Participants were divided into three subgroups based on their Hb, TSAT, and ferritin levels, which reflect their iron status and anemia levels.

The control group consisted of 13 patients with Hb > 11 g/dL, TSAT > 20%, and ferritin > 200 ng/mL. The IDA group included 19 patients with Hb < 9 g/dL, TSAT < 20%, and ferritin < 200 ng/mL. The functional iron deficiency group, representing the intermediate group, included 18 patients with Hb between 9 and 11 g/dL, TSAT < 20%, and ferritin > 200 ng/mL.

Demographic characteristics of HD patients included in the study are presented in detail in Table 2.

**Table 2:** Demographic data of HD patients.

CHARACTERISTIC (*n* = 50)	*N*	%
Gender		
Male	30	60
Female	20	40
Age groups		
18–44	8	16
45–64	15	30
65–84	20	40
>85	7	14
Primary disease, no, %		
Diabetes mellitus	20	27.4
Hypertension	39	53.4
Other	14	19.2
Dialysis duration		
3–12 months	8	16
13–60 months	28	56
> 61 months	14	28
Smoker		
Yes	4	8
No	46	92
Drinking alcohol		
Yes	0	0
No	50	100
Vascular access information		
Arteriovenous fistula (AVF)	26	52
Permanent catheter	24	48
Iron supplement use		
Yes	22	44
No	28	56
Erythropoietin (EPO) use		
Yes	28	56
No	22	44
Angiotensin-converting enzyme inhibitors (ACEI)/Antiotensin II receptor blocker use		
Yes	2	4
No	48	96

AVF, Arteriovenous fistula, ACEI, angiotensin-converting enzyme inhibitors, EPO, erythropoietin.

The patient group consisted of 60% men and 40% women. The majority of patients were aged 45 and over. Hypertension was the most common primary disease at 53.4%, followed by diabetes mellitus at 27.4%. An evaluation of the dialysis duration revealed that 56% of patients had been on dialysis for 13–60 months, and 28% for 61 months or longer. Arteriovenous fistulas (AVF) were the preferred vascular access method in 52% of patients, and 44% received iron supplements, and 56% received erythropoietin (EPO) therapy. Smoking rates were low, alcohol consumption was nonexistent, and only 4% used angiotensin-converting enzyme inhibitors (ACEIs) or angiotensin II receptor blockers as part of antihypertensive treatment.

### 3.2 Comparison of complete blood count and other biochemical parameters among specified groups in HD patients

Laboratory data for the control, FID, and IDA groups are shown in Table 3.

**Table 3:** Comparison of complete blood count and other parameters among specified groups in HD patients.

	Control	FID	IDA	*p*-value
	Median	Median	Median	
	(25th–75th percentile)	(25th–75th percentile)	(25th–75th percentile)	
RBC (10^9/1)	4.015 [3.8575–4.1725]^*^	3.925 [3.365–4.22]	3.27 [2.87–3.42]	<0.01
Hb (mmol/L)	12.25 [11.975–13]^*^	11.5 [10.675–12.775]^†^	9.3 [8.8–10.8]	<0.001
MCV (fL)	93.3 [90.975–94.825]	91 [90.075–92.35]	90 [88.2–94.5]	0.213
MCH (pg)	30.9 [30.2–31.6]	29.35 [28.525–30.925]	29.6 [28.1–31.1]	0.057
TSAT (%)	33.58 [27.2275–61.7175]^║,*^	20.98 [19.2595–23.0325]	16.2 [14.23–20.11]	<0.001
Iron (μmol/L)	66.4 [58.475–70.715]^║,*^	46.56 [41.545–55.05]	35 [27.89–41.9]	<0.001
Ferritin (mg/L)	517.5 [446.795–619.05]	580.815 [334–873.855]	334.05 [241–695]	0.416
ZnPP (μmol/molHb)	7.66 [4.20–9.80]^*^	8.01 [4.54–12.15]^†^	23.25 [9.34–33.13]	<0.05
CRP (mg/L)	9.45 [3.3325–15.925]	12.2 [7.375–23.575]	13.3 [9.88–38.9]	0.106
HCT (%)	37.25 [36.9–37.95]^*^	35.35 [30.875–38.9]^†^	28.6 [27–33.5]	<0.001
MCHC (g Hb/L)	32.7 [32.275–33]	32.35 [32.075–32.925]	32.2 [31.8–32.7]	0.203
MPV (fL)	10.65 [10.075–10.95]^*^	9.35 [9.075–10.45]	9 [8.7–10.1]	*p* < 0.05

CRP, C-reactive protein; HCT, hematocrit; Hb, hemoglobin; HD, hemodialysis; MCH, mean erythrocyte hemoglobin content; MCHC, mean erythrocyte hemoglobin concentration; MCV, mean erythrocyte volume; MPV, mean thrombocyte volume; RBC, red blood cell count; TSAT, transferrin saturation; ZnPP, zinc protoporphyrin.

^║^Pairwise comparison of Kruskal–Wallis test results with the FID group (*p* < 0.05).

^*^Pairwise comparison of Kruskal–Wallis test results with the IDA group (*p* < 0.05).

^†^Pairwise comparison of Kruskal–Wallis test results with the IDA group (*p* < 0.05).

The Shapiro–Wilk test was used to determine whether the data in the specified groups were normally distributed (*p* < 0.05). Differences between the groups were tested with the Mann–Whitney test, and statistically significant differences were found in the red blood cell count (RBC), Hb, TSAT, iron, hematocrit (HCT), mean platelet volume (MPV), and Zinc Protoporphyrin (ZnPP) parameters (*p* < 0.05) (Table 3). RBC, Hb, and HCT levels were found to be statistically significantly lower in the IDA group, reflecting a pronounced anemic picture. TSAT and serum iron levels decreased significantly in the IDA group and in the FID group compared to the control group; however, no significant difference was found in ferritin levels. This suggests that the high ferritin values in the FID group may be related to the inflammatory state. The significantly higher MPV values in the control group indicate possible effects on thrombopoiesis. While there was no statistical significance in MCV and mean erythrocyte hemoglobin content (MCH) levels, the lower levels in the IDA group support microcytic-hypochromic anemia. The findings exhibit that different subtypes of iron deficiency in HD patients lead to distinct changes in the hematological profile, and these parameters reveal significant differences in the differential diagnosis.

Pairwise comparisons of ZnPP levels revealed that ZnPP levels were statistically significantly higher in the IDA group compared to both the FID and control groups. However, no significant difference was detected between the control and FID groups. These findings suggest that ZnPP is significantly increased, particularly in IDA, and may be a potential biomarker in the differential diagnosis.

### 3.3 Evaluation of miRNA-210 expression in HD patients

The significant differences detected in miRNA-210 expression levels in HD patients, because of one-way ANOVA analysis, were further analyzed using Tukey’s multiple comparisons test to determine differences between the groups. Accordingly, the mean difference of the plasma miRNA-210 expression level in the control group was 20.39 *ΔCt* units lower than in the IDA group. The IDA group had significantly up-regulated miRNA-210 expression compared to the control group (*p* = 0.0010).

The mean difference between plasma miRNA-210 expression levels in the control group and the FID group was −1.244, with a confidence interval (95% CI: −14.18 to 11.70). Since the *p*-value was 0.9706, there was no statistically significant difference between the two groups.

The mean difference between plasma miRNA-210 expression levels in the IDA group and the expression levels in the FID group was 19.15 units higher. The IDA group showed upregulation, while the FID group showed downregulation. The 95% CI (7.454 to 30.84) indicated a statistically significant difference between IDA and FID (*p* = 0.0007).

Plasma miRNA-210 *∆*Ct levels of the groups are shown in Table 4.

**Table 4:** Plasma miRNA-210 ∆Ct levels of the groups.

miRNA-210 *∆*Ct	Mean ± SD	Confidence interval (95% CI)	*p*-value
Control and IDA	−20.39 ± 5.29	[−33.19, −7.595]	0.0010***
Control and FID	−1.24 ± 5.35	[−14.18, 11.70]	0.9706
IDA and FID	19.15 ± 4.83	[7.454, 30,84]	0.0007***

IDA, iron deficiency anemia; FID, functional iron deficiency; miRNA, microRNA. ***indicates statistical significance at *p* < 0.001.

**Figure 1:**
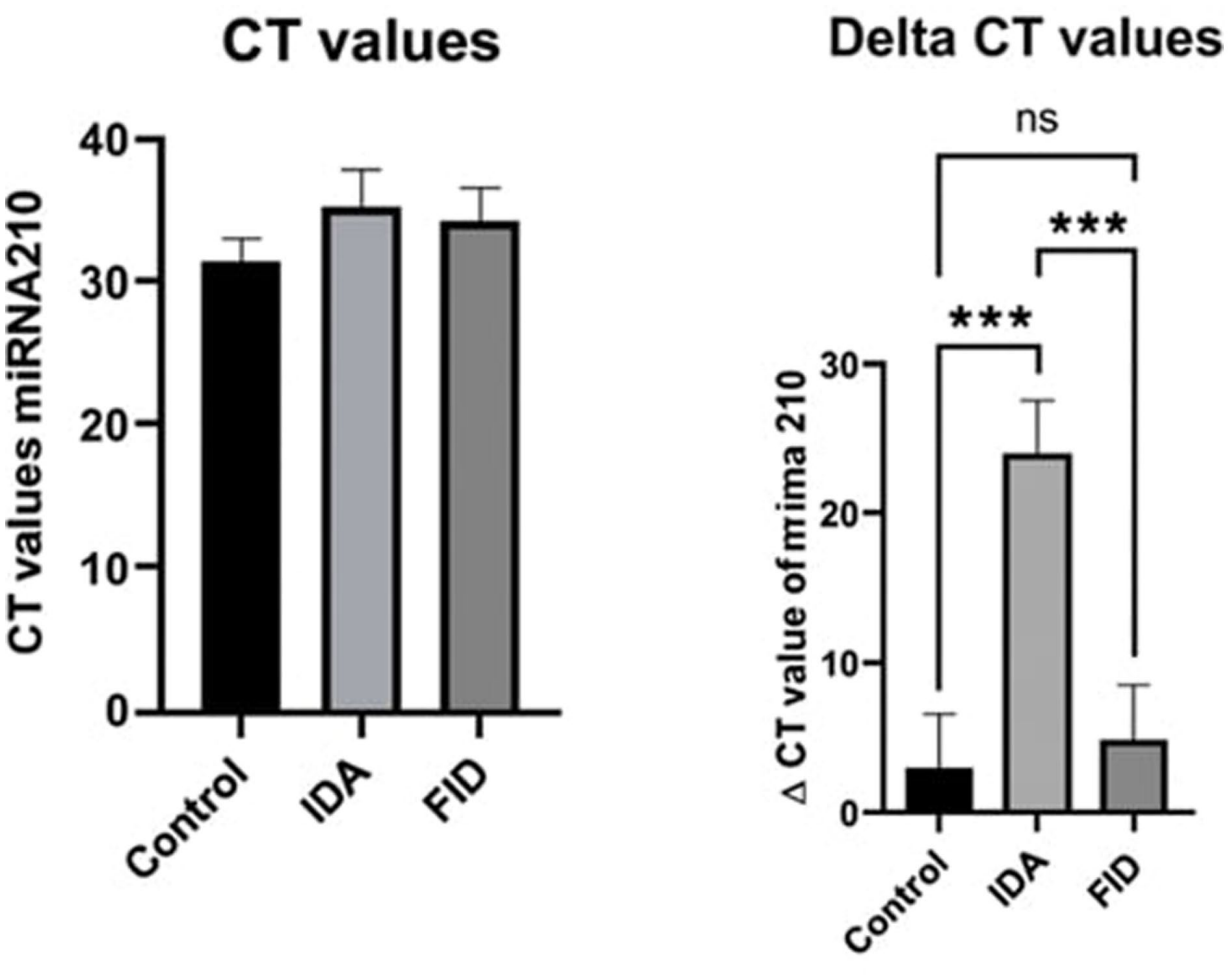
Statistically significant differences in plasma miRNA-210 CT value and ΔCt levels between groups in individuals with HD disease.

Information, including the statistical significance of plasma miRNA-210 CT values and *∆*Ct levels between groups in HD patients, is shown in Figure 1.

**Figure 2:**
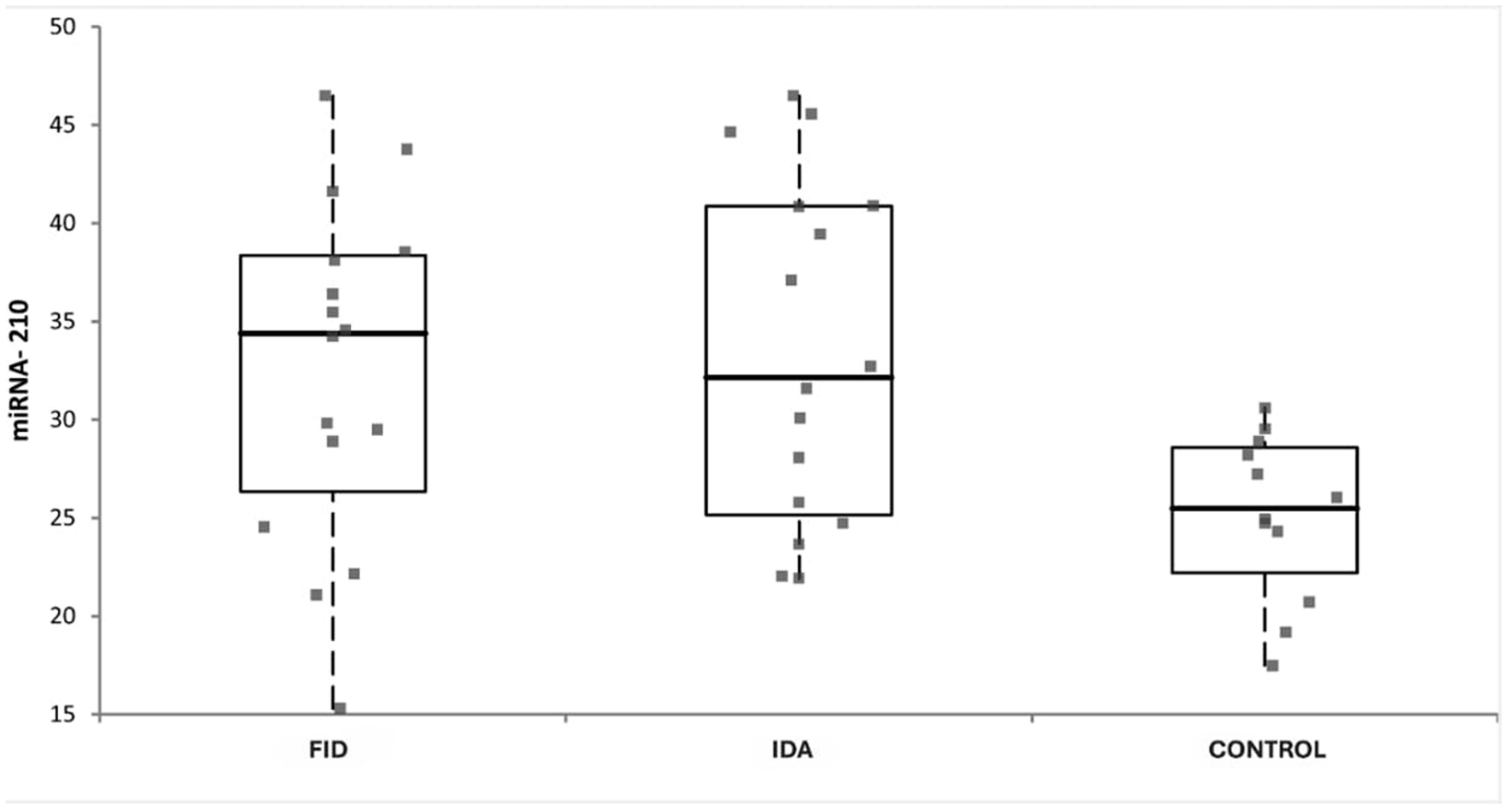
Comparison of miRNA-210 expression levels between groups.

miRNA-210 expression levels in the control, FID, and IDA groups are shown in Figure 2. MiRNA-210 expression levels in the IDA and FID groups are higher and more variable compared to the control group, indicating an increased hypoxic response due to iron deficiency.

**Figure 3:**
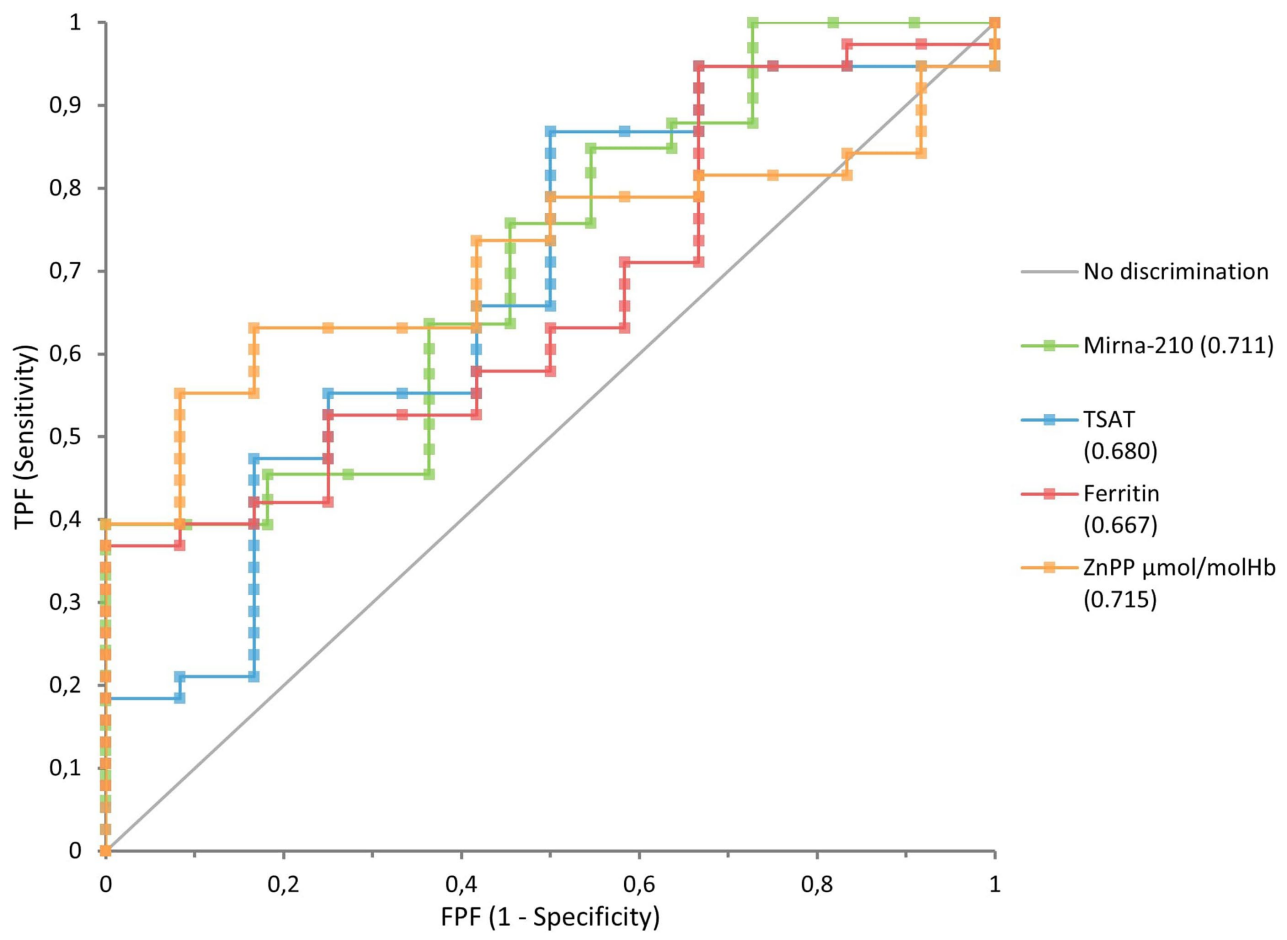
ROC showing the sensitivity and specificity of miRNA-210, TSAT, ZnPP, and Ferritin in distinguishing IDA in chronic HD patients (miRNA, microRNA, ROC, receiver operating characteristic, TSAT, transferrin saturation, ZnPP, zinc protoporphyrin).

To further evaluate the discriminatory potential of IDA in HD patients, Receiver Operating Characteristic (ROC) curve analysis was performed. Accordingly, ROC curves were generated for TSAT, ZnPP, ferritin, and miRNA-210 levels. ROC analysis results revealed that miRNA demonstrated statistically significant diagnostic performance in distinguishing IDA (AUC = 0.711, *p* = 0.0186). This value is higher than that of the traditional parameters TSAT and ferritin, supporting the biomarker potential of this hypoxia-sensitive molecule. The discriminatory power of miRNA-210 is comparable to that of a clinically accepted parameter, such as ZnPP (ZnPP AUC = 0.727, *p* = 0.0017), suggesting that miRNA may have not only supportive but also potentially equivalent diagnostic value. In this context, the biochemical role of ZnPP, reflecting iron metabolism, and miRNA, representing the molecular response associated with cellular stress and hypoxia, suggest that both parameters can be considered complementary biomarkers (Figure 3 and Table 5).

**Table 5:** ROC curve analysis of biomarkers used in determining IDA in HD patients.

Biomarker	AUC	95% CI	*p*-value
ZnPP (μmol/molHb)	0.727	0.585–0.870	*p* < 0.01
miRNA-210	0.711	0.535–0.886	*p* < 0.05
Ferritin (mg/L)	0.657	0.489–0.824	0.0664
TSAT%	0.657	0.474–0.839	0.0925

AUC, area under the curve; CI, confidence interval, FID, functional iron deficiency; IDA, iron deficiency anemia; miRNA, microRNA; ROC, receiver operating characteristic; TSAT, transferrin saturation; ZnPP, zinc protoporphyrin.

**Figure 4:**
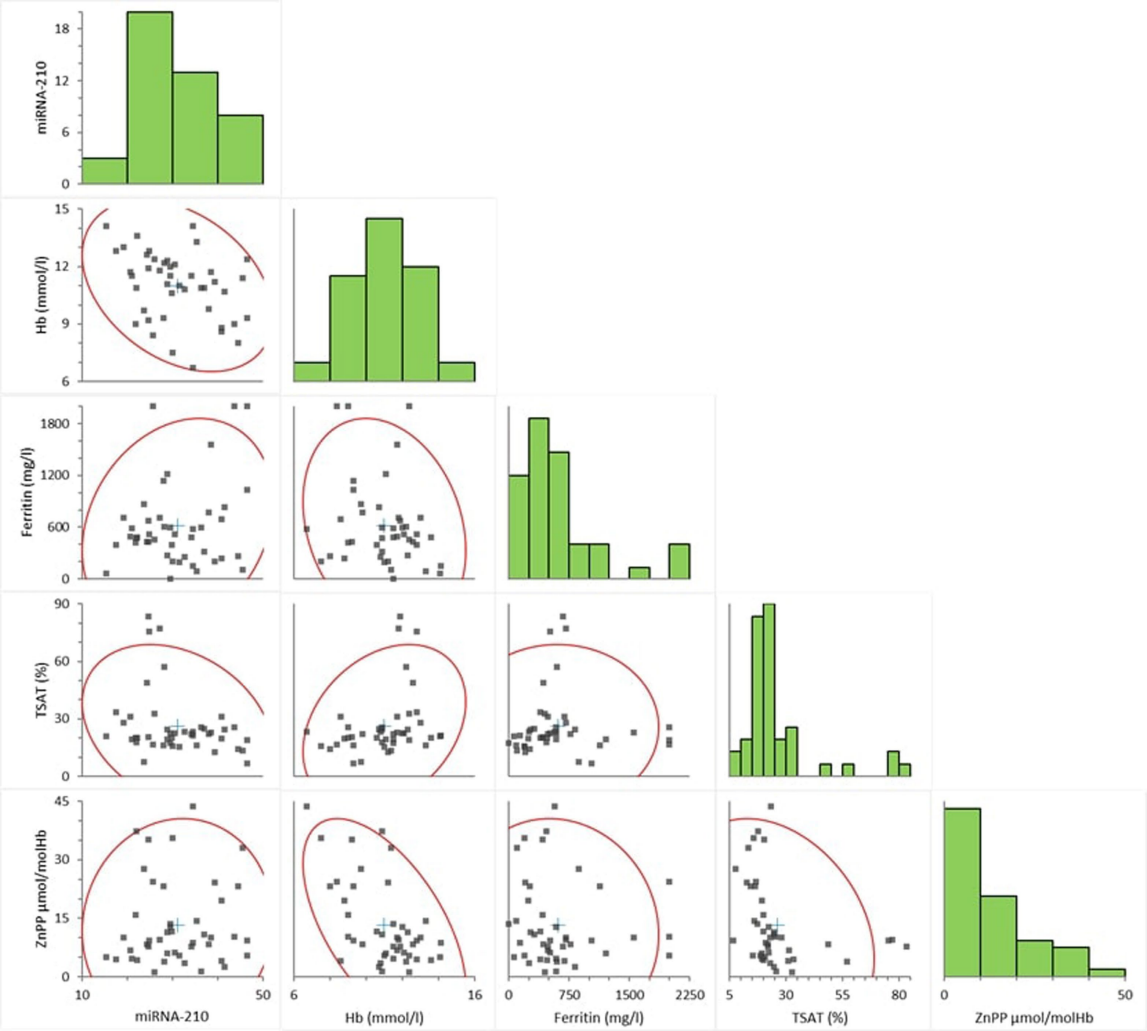
Scatter graph of correlations between miRNA-210, Hb, ferritin, TSAT, and ZnPP in HD patients (Hb, Hemoglobin, miRNA, microRNA; TSAT, transferrin saturation; ZnPP, zinc protoporphyrin).

In the study, the relationships between miRNA-210 levels and hematological and iron metabolism parameters were evaluated using Spearman correlation analysis, and the findings are exhibited in Figure 4.

As a result of the analysis, a negative and statistically significant correlation was found between miRNA-210 and Hb levels (Spearman’s *ρ* = −0.363; *p* = 0.0155). A negative and borderline significant correlation was observed between miRNA-210 and TSAT (*ρ* = −0.269; *p* = 0.0775). In contrast, the relationships of miRNA-210 with ferritin (*ρ* = 0.057; *p* = 0.7118) and ZnPP (*ρ* = 0.062; *p* = 0.6902) levels were not found to be statistically significant.

According to the logistic regression analysis, the relationship between miRNA-210 levels and the presence of anemia was evaluated. The analysis revealed an Odds Ratio (OR) of 1.174 (95% CI: 1.049–1.320) with a *p*-value of 0.022 for miRNA-210. After adjusting for variables such as the number of comorbidities, iron supplementation, EPO use, and ACEI use, miRNA-210 was identified as an independent predictor (Table 6).

**Table 6:** Logistic regression analysis results.

Variable	Beta (*β*)	*p*-value	Odds ratio (OR)	Lower limit (95% CI)	Upper limit (95% CI)
Intercept	−3.917	0.047	–	–	–
miRNA-210	0.117	0.022	1.174	1.049	1.320
Comorbidity (absent/present)	0.754	0.427	2.126	0.331	13.658
Iron supplementation (not using/using)	0.927	0.301	2.527	0.436	14.634
EPO use (not using/using)	1.883	0.036	6.571	1.130	38.199
ACEI use (not using/using)	0.696	0.738	2.007	0.034	119.590

ACEI, angiotensin-converting enzyme inhibitor; CI, confidence interval; EPO, erythropoietin; miRNA, microRNA; OR, odds ratio.

## 4 Discussion

Anemia is defined as Hb levels below the normal range for an individual’s age and sex, while IDA is characterized by decreased body iron stores in addition to anemia and is considered the most common type of anemia globally. IDA is reported to reduce work capacity, negatively impact cognitive function, and impair quality of life in adults (14). Furthermore, numerous studies in the literature indicate that IDA may be associated with chronic diseases (7, 15, 16).

The primary aim of our study was to distinguish between IDA and FID in HD patients by evaluating differences in miRNA-210 expression levels between these two groups. Therefore, as the focus of the study was the comparison of these anemia types, a healthy control group was not included. Although the absence of a healthy control group limits the establishment of reference ranges for circulating miRNA-210 and the generalizability of the findings, our results demonstrate the potential of miRNA-210 levels to differentiate between IDA and FID within the HD population.

The timing of blood sample collection in our study may significantly influence circulating biomarker levels in HD patients, as dialysis procedures can alter the plasma concentrations of certain small molecules, including miRNAs. To minimize this potential confounding effect, all blood samples in our study were collected prior to the initiation of the dialysis session.

In a study conducted by Martino et al., no significant differences were observed in the levels of miR-210 and miR-21 between pre- and post-dialysis samples, and only very small amounts of miRNAs were detected in the dialysate. This finding indicates that circulating miRNAs are largely not removed during dialysis (17). However, other studies have reported marked differences in circulating miRNA profiles across different stages of CKD and in patients undergoing HD. In a study evaluating miR-126 and miR-155 levels, these miRNAs were found to be significantly lower in HD patients compared to both early-stage CKD patients and kidney transplant recipients (18). Furthermore, another study demonstrated that specific miRNAs, such as miR-122, were decreased in individuals with end-stage renal disease prior to dialysis (19).

These findings indicate that while the pre-dialysis sampling strategy partially minimizes potential bias introduced by the dialysis process, the possible effects of dialysis procedures on biomarker levels and the biological variability arising from the intervals between sessions should be carefully considered when interpreting the results. To clarify the impact of these factors, future studies should investigate these issues in greater detail.

In the study, the significantly lower RBC, Hb, and HCT levels in the IDA group confirm the classic anemic picture. The decrease in TSAT and serum iron levels in both the IDA and FID groups compared to the control group suggests that functional iron deficiency can also be observed at the biochemical level. The lack of a significant difference in ferritin levels suggests that the higher values, particularly in the FID group, may be related to inflammation (20). Furthermore, the higher MPV values in the control group may indicate the potential effects of iron deficiency on thrombopoiesis (21). These findings suggest that significant differences in hematological parameters may occur depending on the subtype of iron deficiency in HD patients.

In the literature, ZnPP has been reported as a strong biomarker reflecting iron deficiency and functional iron deficiency. Previous studies have demonstrated that ZnPP may be more reliable than Hb and ferritin in predicting the need for intravenous iron in HD patients and that it plays an especially important role in the assessment of functional iron deficiency. Moreover, large cohort studies have shown that ZnPP accurately reflects iron status, provides high sensitivity and specificity in ROC analyses for the diagnosis of IDA, and yields more reliable results than ferritin and TSAT in the presence of inflammation (22–26).

In our study, ROC analysis was conducted to compare the diagnostic performance of various parameters used to distinguish IDA in HD patients, including TSAT, ferritin, ZnPP, and miRNA-210 levels. The findings demonstrated that miRNA-210 exhibited a statistically significant diagnostic performance (AUC = 0.711, *p* = 0.0186), indicating moderate discrimination for IDA. Accordingly, we interpret miR-210 as a supportive and exploratory biomarker that may complement, but not replace, established measures such as Hb, ferritin, TSAT, and research-stage ZnPP. Its potential clinical utility requires validation in larger, multicenter, longitudinal cohorts with prespecified cut-offs and external replication.

However, it should be noted that ZnPP is not yet endorsed by current clinical guidelines and is not widely used in routine clinical practice. Therefore, the ZnPP findings in our study should be interpreted within an experimental research context, rather than as equivalent to standard clinical biomarkers.

The significant negative correlation between miRNA-210 and Hb levels (*ρ* = −0.363; *p* = 0.0155) suggests that this microRNA may play a regulatory role in erythropoietic processes. This finding is consistent with the literature demonstrating that miRNA-210 is transcriptionally induced via Hypoxia-Inducible Factor 1-alpha (HIF-1α) in response to hypoxia and functions as a key molecular regulator governing cellular adaptation in hypoxic environments (27–29). It is thought that miRNA-210 may reflect adaptive responses that suppress or remodel erythropoiesis, particularly in HD patients exposed to chronic hypoxia. In addition, the fact that the negative correlation between miRNA-210 and TSAT was close to the statistical significance limit (*ρ* = −0.269; *p* = 0.0775) suggests that this miRNA may be associated not only with oxygen-carrying capacity but also with bioactive iron availability. TSAT is considered one of the most clinically sensitive indicators in the evaluation of functional iron deficiency, and in this context, miRNA-210 has the potential to be a biomarker reflecting the molecular interactions between iron bioavailability and erythropoietic stimulation (30).

miRNAs are posttranscriptional regulators that play a critical role at the cellular level in maintaining iron homeostasis. These molecules, which precisely control the cell’s iron uptake, storage, and utilization, are important components of molecular networks that are activated particularly when iron availability is low or oxygen is limited (13, 31–34). At low iron levels, oxygen-carrying capacity decreases with decreased Hb synthesis; on the other hand, in response to hypoxia, iron stores are mobilized and erythropoiesis accelerates. In this process, miRNA-210 stands out as one of the most important hypoxia-sensitive miRNAs. Highly expressed under hypoxic conditions, miRNA-210 has been shown to exert regulatory effects on oxygen balance, iron bioavailability, and cellular adaptation (35).

The importance of miRNA-210 has also been emphasized in various experimental studies on kidney pathophysiology. It has been reported that during processes characterized by oxygen deficiency, such as ischemia/reperfusion injury, this miRNA is expressed at high levels, activating genetic programs that ensure cell survival (36). Furthermore, a study by Douvris et al. (37) demonstrated that miRNAs can be reliably detected in biological fluids such as blood and urine, thus possessing diagnostic potential as non-invasive biomarkers. This view is supported by review studies indicating that miRNA-210 may play a protective role by affecting renal cell adaptation in both acute and chronic kidney diseases (18, 31, 38–43).

The relationship between miRNA-210 and IDA has also been demonstrated in various patient groups. It has been reported that miR-210 levels are significantly increased in individuals with IDA, and this increase correlates with parameters related to iron metabolism (12, 44). It has been emphasized that processes such as ferritin synthesis, iron transport, and utilization are regulated at a post-transcriptional level by miRNA-210 (45). Furthermore, a study in children with IDA found that miRNA-210 is associated not only with iron status but also with the hypercoagulability index (13).

Similarly, increased miRNA-210 levels were observed in patients with *β*-thalassemia/HbE, and this increase has been reported to correlate with their anemic status (46). These findings support the possibility that miRNA-210 may be a sensitive indicator reflecting anemia-related hypoxia. In addition, a study examining the effects of HD on the serum miRNA profile indicated that miRNA-210 levels changed and that these changes may be linked to hypoxia-related cellular adaptation mechanisms (47).

miR-21 has been associated with neointimal hyperplasia, restenosis, and vascular remodeling in HD vascular access pathology. Its increased expression in both vascular tissue and circulation has been linked to access failure. Clinical and translational studies have demonstrated that elevated miR-21 levels may be associated with an increased risk of post-angioplasty restenosis (48). Furthermore, the CKD literature identifies miR-21 as one of the prominent “candidate” miRNAs implicated in kidney disease (49).

miR-210 is a classical hypoxamir regulated through the HIF pathway and influences mitochondrial metabolism and iron–sulfur (Fe-S) cluster biogenesis by repressing Iron–Sulfur Cluster Scaffold Protein 1/2 (ISCU1/2). Through this mechanism, miR-210 establishes a link between hypoxia, iron metabolism, and erythropoiesis (29, 50). Increased miR-210 expression has been observed in cells and tissues in response to hypoxia, and this elevation has been associated with modulation of hypoxic injury in renal cell models (10). In iron deficiency, reduced oxygen delivery to tissues activates HIF-1α, leading to upregulation of miR-210. Sak et al. (12) and Özdemir et al. (13) reported significantly elevated miR-210 levels in patients with IDA. Moreover, Felaco et al. (35) demonstrated that EPO may induce miR-210 expression through the JAK2/STAT5 signaling pathway.

In the context of dialysis, miR-21 and miR-210 were detected only at trace levels in dialysate or ultrafiltrate, indicating that they are not substantially cleared during dialysis. This finding highlights that the timing of pre- and post-dialysis sampling is critical for interpretation, while also suggesting that circulating miRNAs are relatively stable from a technical standpoint (17). In both CKD and HD groups, circulating miRNA profiles, particularly endothelial and inflammatory miRNAs such as miR-126 and miR-155, have been reported to vary according to disease stage (49).

In this context, the use of miR-210 as a biomarker to differentiate IDA from FID in HD patients is pathophysiologically supported by its dual role in reflecting both hypoxia and disrupted iron metabolism.

Consistent with the literature, our study demonstrated that plasma miR-210 levels were significantly elevated in HD patients with IDA. This increase can be interpreted as a molecular reflection of adaptive responses to decreased tissue oxygenation caused by low Hb levels and impaired iron metabolism. Moreover, the observed negative correlations between miR-210 and key hematological parameters such as Hb and TSAT suggest that miR-210 may be sensitive to changes in iron homeostasis and erythropoietic activity.

Previous studies have largely examined anemia using broad and nonspecific definitions, without adequately distinguishing between IDA and FID. These two conditions are biologically distinct: IDA primarily reflects true iron depletion, whereas FID represents a state in which iron stores are sufficient but its mobilization is impaired, often due to underlying inflammation. Failure to distinguish between these phenotypes limits the interpretability of findings. Our study aimed to address this gap by clearly separating IDA and FID within a well-characterized HD cohort. Furthermore, to minimize variability introduced by the clearance effect of dialysis on circulating biomolecules, all blood samples were standardized to the pre-dialysis period.

In this study, miR-210 levels were evaluated alongside both standard clinical biomarkers (Hb, ferritin, TSAT) and the research-based parameter ZnPP. This comprehensive approach enabled us to demonstrate that miR-210 reflects not only a general hypoxic response but also, more specifically, iron deficiency–related hypoxia, providing new insights into anemia mechanisms in HD patients. Unlike previous studies that focused primarily on vascular miRNAs, such as miR-21, our work positions miR-210 within the context of iron metabolism and hypoxia, thereby expanding the current understanding of the biological role of circulating miRNAs in CKD and dialysis populations.

The findings of this study suggest that miR-210 could be integrated into clinical practice as a supportive biomarker, alongside conventional hematological and biochemical parameters. Further research with more extensive causal and functional studies is needed to clarify the role of miR-210 in iron deficiency–related biological processes, which may ultimately contribute to the development of more personalized and targeted strategies for the diagnosis and treatment of anemia.

This study has several limitations that should be considered. First, the relatively small sample size limits both the statistical power and the generalizability of the findings. Additionally, the single-center and cross-sectional design of the study makes it difficult to establish causal relationships and limits the ability to generalize the results to different patient populations.

Since the primary objective of this study was to differentiate between IDA and FID within the HD population, a healthy control group was not included. However, this limitation hinders the establishment of reference ranges for miR-210 and restricts the generalizability of the findings to broader populations. Future studies involving healthy individuals and non-dialysis CKD patients are warranted to facilitate the determination of clinically meaningful cut-off values for miR-210.

Finally, no functional validation was performed at the molecular level. Although this study demonstrated that circulating miR-210 is associated with anemia status in HD patients and negatively correlated with Hb and TSAT levels, downstream molecular targets such as ISCU1/2 or HIF-1α, as well as its functional roles in iron metabolism and hypoxia pathways, were not investigated. Therefore, future multicenter and longitudinal studies incorporating protein-level analyses and cellular models are needed to confirm these findings causally and to better understand the clinical utility of miR-210.

## Data Availability

The datasets generated and analyzed during the current study are available from the corresponding author upon reasonable request. Due to ethical restrictions related to patient data, the datasets are not publicly available.

## References

[1] Gafter-Gvili A Schechter A Rozen-Zvi B. Iron deficiency anemia in chronic kidney disease. Acta Haematol. (2019) 142:44–50. doi: 10.1159/000496492, 30970355

[2] Kazmi S Zarovniaeva V Cortez Perez K Sandhu S Anwar S Mohammed L. Iron-deficiency anemia in chronic kidney disease: a literature review of its pathophysiology, diagnosis, and management. Cureus. (2025) 10.7759/cureus.77598 PMC11830488 39958087

[3] KDIGO. Clinical practice guideline for the evaluation and management of chronic kidney disease. Kidney Int Suppl. (2012) 3:1–150.

[4] Astor BC Muntner P Levin A Eustace JA Coresh J. Association of kidney function with anemia. Arch Intern Med. (2002) 162:1401. doi: 10.1001/archinte.162.12.1401, 12076240

[5] Tawfik YMK Billingsley H Bhatt AS Aboelsaad I Al-Khezi OS Lutsey PL . Absolute and functional iron deficiency in the US, 2017-2020. JAMA Netw Open. (2024) 7:e2433126. doi: 10.1001/jamanetworkopen.2024.33126, 39316402 PMC11423176

[6] Hill NR Fatoba ST Oke JL Hirst JA O’Callaghan CA Lasserson DS . Global prevalence of chronic kidney disease – a systematic review and meta-analysis. PLoS One. (2016) 11:e0158765. doi: 10.1371/journal.pone.0158765, 27383068 PMC4934905

[7] Sany D El Shahawi Y Taha J. Diagnosis of iron deficiency in hemodialysis patients: usefulness of measuring reticulocyte hemoglobin equivalent. Saudi J Kidney Dis Transpl. (2020) 31:1263–72. doi: 10.4103/1319-2442.308335, 33565438

[8] Bavelloni A Ramazzotti G Poli A Piazzi M Focaccia E Blalock W . MiRNA-210: a current overview. Anticancer Res. (2017) 37:7105. doi: 10.21873/anticanres.12107, 29187425

[9] Riester SM Torres-Mora J Dudakovic A Camilleri ET Wang W Xu F . Hypoxia-related microRNA-210 is a diagnostic marker for discriminating osteoblastoma and osteosarcoma. J Orthop Res. (2017) 35:1137–46. doi: 10.1002/jor.23344, 27324965 PMC5413434

[10] Guan Y Song X Sun W Wang Y Liu B. Effect of hypoxia-induced microRNA-210 expression on cardiovascular disease and the underlying mechanism. Oxidative Med Cell Longev. (2019) 2019:1–12. doi: 10.1155/2019/4727283, 31249644 PMC6556335

[11] Dziedzic M Orłowska E Powrózek T Solski J. Role of circulating microRNA in hemodialyzed patients. Postepy Hig Med Dosw (Online). (2016) 70:1362–6. doi: 10.5604/17322693.1227641, 28234233

[12] Sak R Ozbalci D Guchan Alanoglu E Hekimler Ozturk K. Malignancy-related miR-210, mir-373 and let-7 levels are affected in iron deficiency anemia. Afr Health Sci. (2023) 23. doi: 10.4314/ahs.v23i3.30, 38357103 PMC10862612

[13] Özdemir ZC Düzenli Kar Y Bör Ö. Whole blood miR-210, miR-122, miR-223 expression levels and their relationship with Iron status parameters and hypercoagulability indices in children with Iron deficiency Anemia. J Pediatr Hematol Oncol. (2021) 43:e328–35. doi: 10.1097/MPH.0000000000002127, 33710119

[14] Joosten E. Iron deficiency anemia in older adults: a review. Geriatr Gerontol Int. (2018) 18:373–9. doi: 10.1111/ggi.13194, 29094497

[15] Fishbane S Singh AK. Iron metabolism in patients with chronic kidney disease. Semin Nephrol. (2009) 29:57–65.

[16] Bager P Befrits R Wikman O Lindgren S Moum B Hjortswang H . The prevalence of anemia and iron deficiency in IBD outpatients in Scandinavia. Scand J Gastroenterol. (2011) 46:304–9. doi: 10.3109/00365521.2010.533382, 21073374

[17] Martino F Lorenzen J Schmidt J Schmidt M Broll M Görzig Y . Circulating microRNAs are not eliminated by hemodialysis. PLoS One. (2012) 7:e38269. doi: 10.1371/journal.pone.0038269, 22715378 PMC3371001

[18] Franczyk B Gluba-Brzózka A Olszewski R Parolczyk M Rysz-Górzyńska M Rysz J. miRNA biomarkers in renal disease. Int Urol Nephrol. (2022) 54:575–88. doi: 10.1007/s11255-021-02922-7, 34228259 PMC8831254

[19] Rivoli L Vliegenthart ADB de Potter CMJ van Bragt JJMH Tzoumas N Gallacher P . The effect of renal dysfunction and haemodialysis on circulating liver specific miR-122. Br J Clin Pharmacol. (2017) 83:584–92. doi: 10.1111/bcp.13136, 27650800 PMC5306495

[20] Ganz T Nemeth E. Iron homeostasis in host defence and inflammation. Nat Rev Immunol. (2015) 15:500–10. doi: 10.1038/nri3863, 26160612 PMC4801113

[21] Cho SY Yang JJ Suh J-T Lee W-I Lee HJ Park TS. Mean platelet volume/platelet count ratio in anemia. Platelets. (2012) 24:244–5. doi: 10.3109/09537104.2012.684734, 22646052

[22] Zimmermann MB Molinari L Staubli-Asobayire F Hess SY Chaouki N Adou P . Serum transferrin receptor and zinc protoporphyrin as indicators of iron status in African children. Am J Clin Nutr. (2005) 81:615–23. doi: 10.1093/ajcn/81.3.615, 15755831

[23] Baldus M Salopek S Möller M Schliesser J Klooker P Reddig J . Experience with zinc protoporphyrin as a marker of endogenous iron availability in chronic haemodialysis patients. Nephrol Dial Transplant. (1996) 11:486–91. doi: 10.1093/oxfordjournals.ndt.a027316 8710158

[24] Braun J Hammerschmidt M Schreiber M Heidler R Hörl WH. Is zinc protoporphyrin an indicator of iron-deficient erythropoiesis in maintenance haemodialysis patients? Nephrol Dial Transplant. (1996) 11:492–7. doi: 10.1093/oxfordjournals.ndt.a027317 8671820

[25] Fishbane S Lynn RI. The utility of zinc protoporphyrin for predicting the need for intravenous iron therapy in hemodialysis patients. Am J Kidney Dis. (1995) 25:426–32. 7872320 10.1016/0272-6386(95)90104-3

[26] Leventi E Aksan A Nebe CT Stein J Farrag K. Zinc protoporphyrin is a reliable marker of functional iron deficiency in patients with inflammatory bowel disease. Diagnostics. (2021) 11:366. doi: 10.3390/diagnostics11020366, 33670067 PMC7926353

[27] Huang X Le QT Giaccia AJ. MiR-210 – micromanager of the hypoxia pathway. Trends Mol Med. (2010) 16:230–7. doi: 10.1016/j.molmed.2010.03.004, 20434954 PMC3408219

[28] Camps C Buffa FM Colella S Moore J Sotiriou C Sheldon H . hsa-miR-210 is induced by hypoxia and is an independent prognostic factor in breast cancer. Clin Cancer Res. (2008) 14:1340–8. doi: 10.1158/1078-0432.CCR-07-1755, 18316553

[29] Chan SY Zhang YY Hemann C Mahoney CE Zweier JL Loscalzo J. MicroRNA-210 controls mitochondrial metabolism during hypoxia by repressing the Iron-sulfur cluster assembly proteins ISCU1/2. Cell Metab. (2009) 10:273–84. doi: 10.1016/j.cmet.2009.08.015, 19808020 PMC2759401

[30] Weiss G Goodnough LT. Anemia of chronic disease. N Engl J Med. (2005) 352:1011–23. doi: 10.1056/nejmra041809, 15758012

[31] Hui X Al-Ward H Shaher F Liu CY Liu N. The role of miR-210 in the biological system: a current overview. Hum Hered. (2019) 84:233–9. doi: 10.1159/000509280, 32906127

[32] Castoldi M Muckenthaler MU. Regulation of iron homeostasis by microRNAs. Cell Mol Life Sci. (2012) 69:3945–52. doi: 10.1007/s00018-012-1031-4, 22678662 PMC11114850

[33] Davis M Clarke S. Influence of microRNA on the maintenance of human iron metabolism. Nutrients. (2013) 5:2611–28. doi: 10.3390/nu5072611, 23846788 PMC3738991

[34] Li J Ma L Yu H Yao Y Xu Z Lin W . MicroRNAs as potential biomarkers for the diagnosis of chronic kidney disease: a systematic review and meta-analysis. Front Med Lausanne. (2022). 8:782561. doi: 10.3389/fmed.2021.782561 35198569 PMC8860181

[35] Felaco P Felaco M Franceschelli S Ferrone A Gatta DMP Speranza L . Erythropoietin induces miRNA-210 by JAK2/STAT5 signaling in PBMCs of end-stage renal disease patients. FEBS J. (2020) 287:5167–82. doi: 10.1111/febs.15302 32196922

[36] Zawada AM Rogacev KS Müller S Rotter B Winter P Fliser D . Massive analysis of cDNA ends (MACE) and miRNA expression profiling identifies proatherogenic pathways in chronic kidney disease. Epigenetics. (2014) 9:161–72. doi: 10.4161/epi.26931 24184689 PMC3928179

[37] Douvris A Viñas JL Akbari S Tailor K Lalu MM Burger D . Systematic review of microRNAs in human acute kidney injury. Ren Fail. (2024) 46:2419960. doi: 10.1080/0886022X.2024.2419960 39477814 PMC11533245

[38] Watany MM Hagag RY Okda HI. Circulating miR-21, miR-210 and miR-146a as potential biomarkers to differentiate acute tubular necrosis from hepatorenal syndrome in patients with liver cirrhosis: a pilot study. Clin Chem Lab Med (CCLM). (2018) 56:739–47. doi: 10.1515/cclm-2017-0483, 29303765

[39] Petrozza V Costantini M Tito C Giammusso LM Sorrentino V Cacciotti J . Emerging role of secreted miR-210-3p as potential biomarker for clear cell renal cell carcinoma metastasis. Cancer Biomark. (2020) 27:181–8. doi: 10.3233/CBM-190242, 31771042 PMC12662287

[40] Samaan S Khella HWZ Girgis A Scorilas A Lianidou E Gabril M . miR-210 is a prognostic marker in clear cell renal cell carcinoma. J Mol Diagn. (2015) 17:136–44. doi: 10.1016/j.jmoldx.2014.10.005, 25555365

[41] Iwamoto H Kanda Y Sejıma T Osakı M Okada F Takenaka A. Serum miR-210 as a potential biomarker of early clear cell renal cell carcinoma. Int J Oncol. (2014) 44:53–8. 24212760 10.3892/ijo.2013.2169

[42] McCormick RI Blick C Ragoussis J Schoedel J Mole DR Young AC . miR-210 is a target of hypoxia-inducible factors 1 and 2 in renal cancer, regulates ISCU and correlates with good prognosis. Br J Cancer. (2013) 108:1133–42. doi: 10.1038/bjc.2013.56, 23449350 PMC3619073

[43] van Lieshout TS Klerks AK Mahic O Vernooij RWM Eisenga MF van Jaarsveld BC . Comparative iron management in hemodialysis and peritoneal dialysis patients: a systematic review. Front Nephrol. (2024) 4:1488758. doi: 10.3389/fneph.2024.1488758 39664943 PMC11631840

[44] Pal JK Sur S Mittal SPK Dey S Mahale MP Mukherjee A. Clinical implications of miRNAs in erythropoiesis, anemia, and other hematological disorders. Mol Biol Rep. (2024) 51:1064. doi: 10.1007/s11033-024-09981-w, 39422834

[45] Yoshioka Y Kosaka N Ochiya T Kato T. Micromanaging iron homeostasis. J Biol Chem. (2012) 287:34110–9. doi: 10.1074/jbc.M112.356717, 22896707 PMC3464520

[46] Siwaponanan P Fucharoen S Sirankapracha P Winichagoon P Umemura T Svasti S. Elevated levels of miR-210 correlate with anemia in β-thalassemia/HbE patients. Int J Hematol. (2016) 104:338–43. doi: 10.1007/s12185-016-2032-0, 27272941

[47] Trzybulska D Eckersten D Giwercman A Christensson A Tsatsanis C. Alterations in serum microRNA profile during hemodialysis - potential biological implications. Cell Physiol Biochem [Internet]. 2018;46:793–801 29627823 10.1159/000488737

[48] Wu CC Chen LJ Hsieh MY Lo CM Lin MH Tsai HE . MicroRNA-21 and venous neointimal hyperplasia of dialysis vascular access. Clin J Am Soc Nephrol. (2018) 13:1712–20. doi: 10.2215/CJN.02410218, 30242025 PMC6237050

[49] Peters LJF Floege J Biessen EAL Jankowski J van der Vorst EPC. MicroRNAs in chronic kidney disease: four candidates for clinical application. Int J Mol Sci. (2020) 21:6547. doi: 10.3390/ijms21186547, 32906849 PMC7555601

[50] Chen Z Li Y Zhang H Huang P Luthra R. Hypoxia-regulated microRNA-210 modulates mitochondrial function and decreases ISCU and COX10 expression. Oncogene. (2010) 29:4362–8. doi: 10.1038/onc.2010.193, 20498629

